# Empowering diversity: striving for inclusivity by leveraging the American Medical Informatics Association’s “For Your Informatics” Podcast

**DOI:** 10.1093/jamiaopen/ooae072

**Published:** 2024-09-17

**Authors:** Karmen S Williams, Vivian Hui, Mindy Ross, Davina J Zamanzadeh, Vickie Nguyen, Zubin A Khan, Wendy W Chapman, Kai-Lin You, Anita Murcko, Leyla Warsame, Wendy M Ingram, Tiffany Harman, Adela Grando

**Affiliations:** Department of Health Policy and Management, Graduate School of Public Health and Health Policy, City University of New York, New York, NY 10027, United States; Center for Smart Health, School of Nursing, The Hong Kong Polytechnic University, Kowloon, Hong Kong; Health and Community Systems, School of Nursing, University of Pittsburgh, Pittsburgh, PA 15213, United States; Division of Pediatric Pulmonology, University of California, Los Angeles, CA 90095, United States; Department of Computer Science, University of California, Los Angeles, CA 90095, United States; UX Consultant, Houston, TX 77002, United States; Salt Lake County Health Department, Salt Lake City, UT 84111, United States; Centre for Digital Transformation of Health, University of Melbourne, Parkville Victoria 3010, Australia; School of Nursing, University of Pittsburgh, Pittsburgh, PA 14213, United States; College of Health Solutions, Arizona State University, Phoenix, AZ 85004, United States; Housecalls, Northwell Health Solutions, Manhasset, NY 11030, United States; Dragonfly Mental Health, Bradenton, FL 34205, United States; Health Information Systems, Solventum, Murray, UT 84123, United States; College of Health Solutions, Arizona State University, Phoenix, AZ 85004, United States

**Keywords:** podcast, diversity, rebranding, social media

## Abstract

**Importance:**

Starting in 2018, the ‘Women in American Medical Informatics Association (AMIA) Podcast’ was women-focused, in 2021 the podcast was rebranded and relaunched as the “For Your Informatics Podcast” (FYI) to expand the scope of the podcast to include other historically underrepresented groups. That expansion of the scope, together with a rebranding and marketing campaign, led to a larger audience and engagement of the AMIA community.

**Objectives:**

The goals of this case report are to characterize our rebranding and expanding decisions, and to assess how they impacted our listenership and engagement to achieve the Podcast goals of increasing diversity among the Podcast team, guests, audience, and improve audience engagement.

**Materials and Methods:**

This descriptive case study is focused on the FYI Podcast team’s processes to develop a revised mission, vision, and values, increase the diversity of guests, augment listenership through social media, and track the reach through the number of followers, downloads, and impressions.

**Results:**

As of December 2023, 35 FYI Podcast episodes are available with 685 social media followers, over 20 000 downloads, and nearly 145 000 impressions. In addition to introductions to informatics and loyal listeners within AMIA, the FYI Podcast episodes have been used by students as teaching material in a graduate biomedical informatics curriculum, and as introductory material for student clubs and programs.

**Discussion:**

The Podcast relaunching led to 98% of guests from underrepresented groups and growth in listenership by 329% since May 2021.

**Conclusion:**

The FYI Podcast supports AMIA’s diversity mission, and gives voices to underrepresented groups, engages the clinical informatics community in critical conversations on justice, equity, diversity and inclusion, and supports education.

## Introduction

Sharing information can be a powerful catalyst for scientific progress.^1^ Individual’s collective stories have the potential to uncover novel insights and shape the future of healthcare. In recent decades, podcasts have grown in popularity for information sharing.^2^^,^^3^ An estimated 205.5 million people (62%) in the US have listened to a podcast at least once in their lifetime, and over 144 million listen monthly.^4^ According to Edison Research in 2022, an estimated 59% White, 46% of females, 16% African American, 16% Latino, 3% Asian, and 6% other ethnicities, listen to podcast on a regular basis.^4^^,^^5^ The format of podcasting allows accessibility and convenience to listen to informative and engaging content while performing tasks in daily routines, such as commuting or exercising.^6^

Embracing this powerful form of digital storytelling, the Women in the American Medical Informatics Association (AMIA) organized as an affinity group and produced a podcast committed to “informing and inspiring the informatics community toward action around opportunities for women in AMIA to improve health and healthcare.”^7^ The Women in AMIA (WIA) Podcast was well-received and was awarded funding through WIA Leadership Program Seed Grants in 2020 to continue to mature the Podcast through expanded content and listenership.

Mainstream informatics content and perspectives were already represented, so the WIA Podcast expanded the mission to promote diversity and inclusion to ultimately lead to more innovative and creative solutions, positive impacts on the healthcare industry, and address the existing disparities in the health informatics field.^8–10^ In 2021, the Podcast was rebranded and relaunched as the For Your Informatics (FYI) Podcast^11^ to “explore the limitless world of biomedical informatics”. The aim of this case study is to describe the process of starting from a women-focused podcast (WIA podcast), to expand the scope of the podcast (FYI Podcast) to include guests from historically underrepresented groups (eg, African Americans and Latinx individuals). That expansion of the scope, together with a rebranding and marketing campaign, led to a larger audience and engagement of the AMIA community. The goals of this case report are to characterize our rebranding and expanding decisions, and to assess how they impacted our listenership and engagement to achieve the Podcast goals: (1) a more diverse (gender, age, ethnicity/race, and expertise) Podcast team, (2) more Podcast guests from historically underrepresented groups, (3) increased Podcast audience, (4) higher audience engagement, and (5) use of Podcast content for educational purposes.

## Methods

The FYI Podcast team compiled research on podcasts, comments from listeners, and reviewed the current processes to rebrand the podcast. We evaluated from this information the needs of the team to produce quality episodes, such as equipment, expertise, and measures.

### Mission, audience, and content development

Building on the mission of the WIA Podcast, the FYI Podcast rebranding process included reviewing and revising the mission, vision, values, and target audience. Modifications were determined through collaborative discussion, decision making, and audience engagement. The Podcast team addressed the look and feel of the Podcast by creating a new logo and original theme music for the Podcast’s intro and outro.

WIA Podcast’s guests were identified through suggestions from the WIA committees by having dedicated social media sites and email addresses; the guest recommendations for the FYI Podcast come from the informatics community and beyond. FYI Podcast also partnered with other podcasts such as the Association of Clinical Informatics Fellows—ACIF Go-Live Podcast^12^ and the Association of Computing Machinery ByteCast^13^ with a shared vision to increase content and reach.

The episode format is a semi-structured interview with one or more guests, occasionally with an introduction and narration by the Podcast team or invited guests. The interviews are conducted by phone, teleconferencing platforms such as Zoom, and in-person at conferences, including sessions with a live audience at the AMIA Annual Symposiums.

### Equipment, costs, and platforms

Podcast production quality is highly dependent upon the caliber of the equipment used for recording and editing. Recording and editing the Podcast for distribution needs the following: headphones, a microphone, remote teleconference software, an audio recording application software, and a digital audio editor. The FYI Podcast team consulted with audio experts and materials to identify multiple options to select the best methods.

Operation of the FYI Podcast is roughly $5000 per year. The costs include hardware (eg, microphones), software (eg, media design), student stipends, editor payments, mailing, printing, and materials for booths at conferences. To offset these expenses, the FYI Podcast team has relied on in-kind donations, support from AMIA and WIA, volunteers, sponsorships (eg, Emory Nell School of Nursing Sponsorship), and grants (eg, The WIA Leadership Program Seed Grants).

FYI Podcast is available through the AMIA.org website,^14^ iTunes,^15^ Spotify,^16^ Amazon Music,^17^ SoundCloud,^18^ and iHeartRadio.^19^ In 2022, the FYI joined the Public Health Podcast and Media Network’s Directory as its first informatics-based podcast.^20^^,^^21^

### FYI podcast team and episode release process

The team held an open call to the AMIA Student Working Group, WIA, and the general AMIA membership to invite participants for hosts, social media ambassadors, and advisors. The Podcast team consists of a director (K.W.), advisors (M.R., A.G., T.H., V.N., Z.K.), treasurer (D.Z.), hosts (L.W., A.M., W.I., A.G.), editor (D.I.), social media ambassadors (V.H., K.Y.), and an AMIA staff liaison (K.T.). The director oversees the Podcast operations, organizes guests and episode direction, and consults with team on decisions. The advisors are a steering committee who attend meetings and participate in decisions-making discussions. The treasurer is responsible for managing the expenses required to produce episodes and the operating costs of the Podcast overall, such as marketing and student awards. The hosts meet with guests and record episodes. The editor trims the audio files and integrates music, introductions and closing of each episode. The social media ambassadors are responsible for digital media creation and posting, data analytics, and monitor end-user feedback. The AMIA staff liaison helps with website updates, logistics at conferences, and advocates for the Podcast within AMIA.

Once the Podcast team has decided on an episode, and it is recorded, the host transmits the audio files to the editor. The edited episode is reviewed by the director, host, and guests for any final edits, and sent to the AMIA staff liaison to be posted on all podcast platforms. The episodes are promoted by the social media ambassadors. Additionally, the episodes are transcribed and posted on the website, and when available, a list of resources and books also.

### Social media and analytics

The WIA Podcast generally used individual accounts to share information about episodes and content. For the rebranding, FYI podcast needed separate branded sites that reflected both individuality and cohesiveness with the current AMIA brand. To gauge effectiveness towards the new goals, FYI Podcast needed a systematic and regular collection of measures to assist with future content, post types, and feedback from listeners. Measures included listeners, followers, audience growth rates, visitors, demographics, labels and hashtag counts, and engagement (such as likes, shares, and comments).

Twitter.com^22^ and LinkedIn.com^23^ pages on personal accounts were originally used to promote the WIA Podcast. For the FYI Podcast, a social media team was established, and 4 dedicated and branded social media channels, Twitter,^24^ LinkedIn,^25^ Instagram,^26^ and Facebook,^27^ are used for promotion.

Canva.com^28^ is used to create graphics for branding and social media campaigns, and Buffer.com is used to manage and schedule social postings. Social media calendars and spreadsheets are used to manage Podcast posts and track the progress and roll-out of the episodes to help maximize reach to the target audience. Buffer.com^29^ is also used for social media analytics and measures, such as audience growth rate, new follower count, follower-following ratio, visitors, demographics, labels and hashtags, and engagement (eg, likes, shares, comments, clicks, etc.) per post. Each social media site is individually monitored and aggregated across their shared metrics.

## Results

### Podcast team

The first goal of the FYI Podcast was achieved with a more diverse Podcast Team that included different age groups (mid-20s to mid-60s), all stages of career development (including for first-time students to Associate Professors and Directors), representatives of both academia and industry, minority groups (African-American and Latinx) and was not limited to women as before.

### Mission and guests

We achieved the second goal, with 11% more Podcast guests from historically underrepresented racial and ethnic groups (an increase from 38% to 49%). In addition to the original WIA Podcast’s intent, the updated FYI Podcast mission focuses on interviewing and attracting historically underrepresented groups into the biomedical informatics pipeline, creating podcasts that can be used in multipurpose settings, and supporting education. The values of the FYI Podcast are inclusivity, diversity, leadership and education, and the vision is to “share the limitless world of biomedical informatics and its diverse voices to inspire greatness in the field.”

The Podcast’s guests and target audience are biomedical informatics professionals, all levels of students, and those interested in learning more about biomedical informatics careers, research, and practice.

### Episodes production and audience

**Figure 1.**
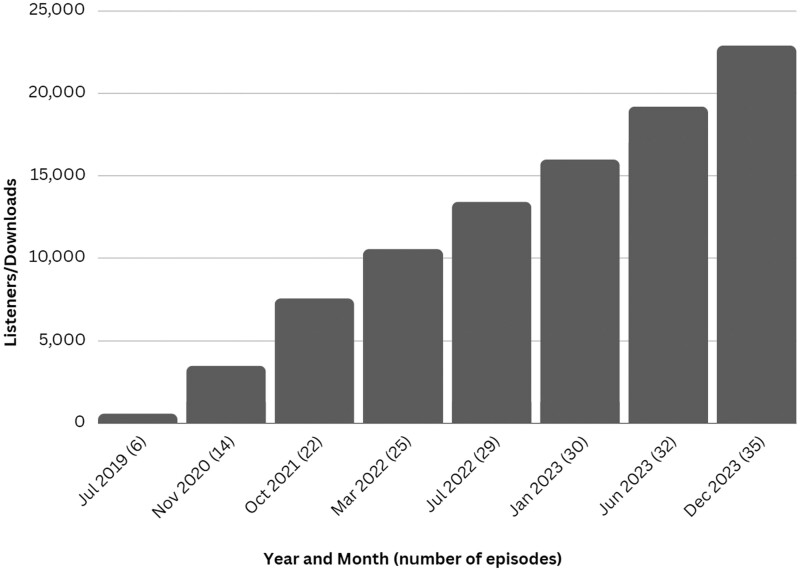
Number of unique downloads (from 2019 to 2023).

**Figure 2.**
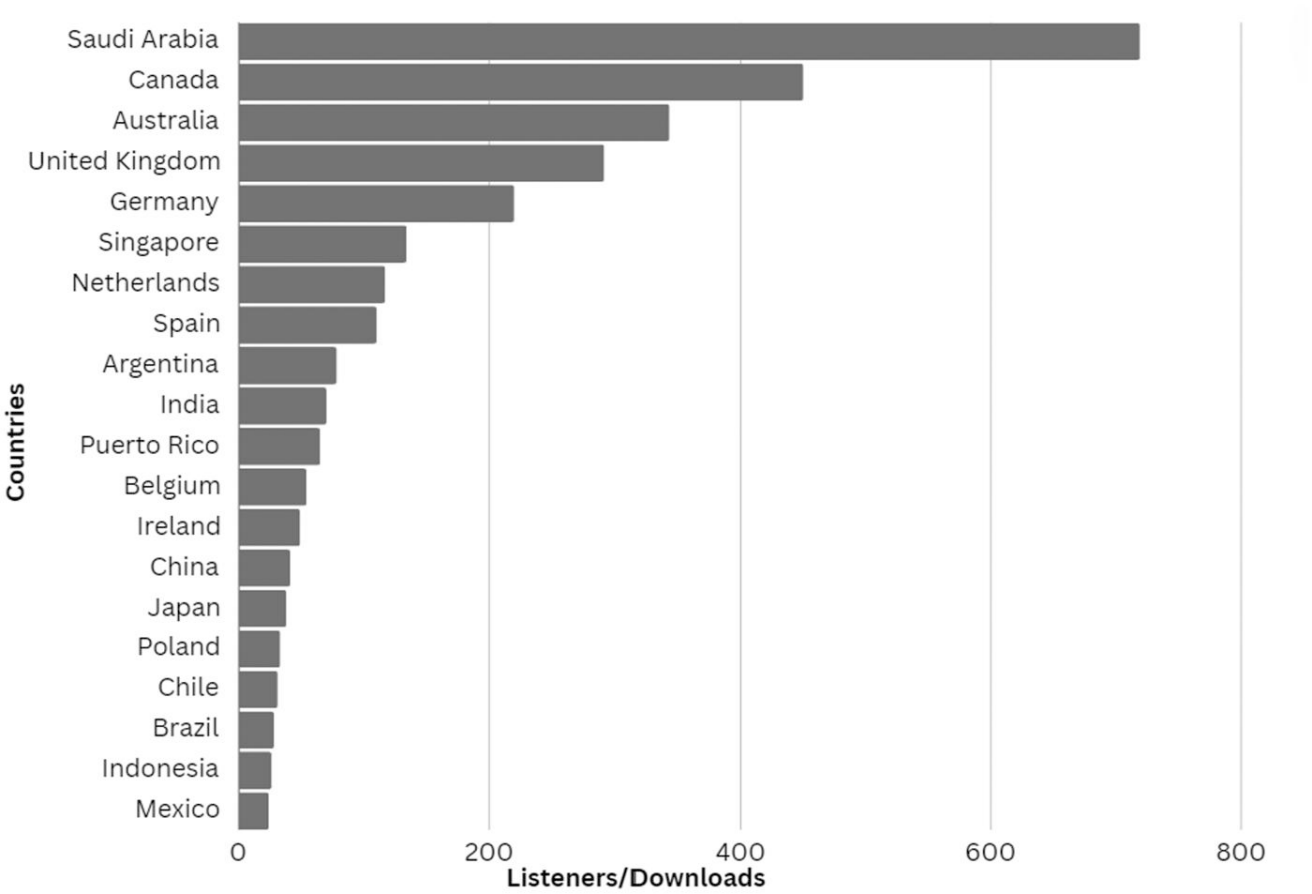
Top 20 countries with unique downloads as of December 2023 [US = 18 372].

The third goal of the FYI podcast was achieved. Since May of 2021, FYI Podcast has seen a 329% increase in listeners. Overall, 35 Podcast episodes with 54 guests are available, including 15 that were produced before the rebranding. The FYI Podcast had over 20 000 unique downloads in 78 countries, with the majority (81%; 15 578) of listenership in the United States (Figures 1 and 2).

Episodes are an average of 34.7 min (SD = 12.8). Topics have included global informatics, gender pay gap in medicine, diversity in leadership, informatics career paths, mentoring and networking, Journal of the American Medical Informatics Association (JAMIA), AMIA student group, and the Forgotten No More Series that highlights the overlooked informatics contributions of women (**Table S1**).

Approximately 15% of listenership was from non-US countries, the speakers and topics have expanded, and the downloads by students and more than just women have increased based on social media testimonials.

### Social media coverage

The fourth goal of the FYI Podcast was achieved. As of December 2023, the FYI Podcast had 700 followers across the four social media platforms. The most followed platform is Twitter (*n* = 330, 48%), LinkedIn (*n* = 293, 42%) is second, Instagram (*n* = 68, 10%) third, and the least followed platform is Facebook (*n* = 9, 1.3%) (Table 1).

**Table 1.** Social media impression and engagement statistics until December 2023.

Platforms	Followers (*n*, %)^a^	Total retweets/reposts^a^	Total replies^a^	Impressions^a^	Posts	Clicks/reach^a^	Likes^a^
**Twitter**	330 (48%)	422	25	126 342	209	269	858
**LinkedIn**	293 (42%)	N/A	N/A	8665	65	235	N/A
**Instagram**	68 (10%)	N/A	28	5845	206	4318	318
**Facebook**	9 (1.2%)	2	2	1052	225	452	5

a “Followers” indicate the number of accounts that have subscribed or follow to the social media page. “Total Retweets and Reposts” are the number of times the post has been shared to a new page or profile. The “total replies” are the number of times comments are made on the posts. “Impressions” indicate the number of times the post was seen. “Clicks/Reach” is the number of times the posts are actually clicked. “Likes” are the number of times the posts received a like or reaction.

There are also over 141 904 impressions recorded across all social media platforms. On Twitter, the top performing hashtags were #ClinicalInformatics, #CIFellowships, and #Leaders with average impression of 3813 and engagement of 1.4%. On LinkedIn, it was #Diversity, #Equity, #Inclusion, #foryourinformatics and #digitalhealth with the highest impression. On Instagram, #FYIFavoriateEpisodes obtained the highest reach while #WeAreFYInformatics with an average impression of 99 and 4.76% engagement rate. Lastly, Facebook yielded the least average impression of 1052.

### Educational use

The fifth goal was achieved. The FYI podcast is an impactful educational tool. It has been incorporated into the graduate biomedical informatics curriculum at Arizona State University (ASU), and population health informatics courses at the City University of New York (CUNY). Since 2020, nearly 130 ASU and 100 CUNY masters and doctoral students have listened to FYI Podcast episodes as part of their core health informatics introductory courses. Topical podcasts have been incorporated as background material for coursework in several courses as part of assignments and/or extra credit. Students have expressed excitement about the timely “real world” applications of academic concepts covered by the podcasts such as the 21st Century Cures Act: “As I listened to the host’s questions and the guests’ insight, I felt more knowledgeable about a topic that I had been learning about for the past 6 months. I feel grateful to have listened.”

Additionally, FYI Podcast have also inspired student-led clubs like the ASU’s Students of BMI, as well as provided an informatics introduction to high school and undergraduate programs.^9^^,^^30^ For example, in the past 5 years, the AMIA First Look Program has used the Podcast in their informatics introduction to over 120 undergraduate students.

## Discussion

In the United States, women and historically marginalized ethnoracial groups have been underrepresented in the field of medical informatics.^8^ To raise awareness and address the issue, the WIA developed a podcast as an approach to increase success and continued pipeline of diversity in the field. This case report highlights the efforts and strategies to successfully build and grow this vehicle in medical informatics.

The FYI Podcast showcases information and overlooked perspectives within the medical informatics community that promotes and can attract professionals in underrepresented and historically minoritized groups. A diverse community leads to greater innovation as it makes available much broader perspectives and leads to improved outcomes with reduced biases.^1^^,^^31^ Increasing diversity is an ongoing strive, but the FYI Podcast has seen movement in these efforts. Additionally, plans to partner with other informatics and health technology groups that cater to historically underrepresented groups (eg, Grace Hopper Women in Computing) will improve diverse guests and listenership.

Logistically, academia is typically under-resourced^32^ and the FYI Podcast team is comprised of medical informatics professionals with full-time responsibilities in different locations. Podcasting provides an efficient, asynchronous production allowing for a high-quality product despite limited bandwidth and resources. In addition, the medium is a well-aligned format for consumption by the busy target audience.

Limitations of this work include a podcast team comprised primarily of volunteers, and a lack of consistent funding. The team began this work due to interests in increasing diversity in the medical informatics pipeline through the power of social media and digital mediums but did not have formal expertise nor formal consistent funding in this domain.

Future endeavors aim to continue the expansion of the FYI Podcast’s reach with increased advertising and sponsorship. The Podcast team plans to further track listenership and advertising, and measure entry into the field, such as tracking AMIA members who cite the Podcast as influential in their career or membership choice.

## Conclusion

The FYI Podcast supports AMIA’s diversity mission by giving voices to historically underrepresented groups by increasing the diversity of the speaker and the audience and supporting medical informatics education. The FYI Podcast seeks to engage the biomedical informatics community in critical conversations about justice, equity, diversity and inclusion with “teachable moments” for learners at all levels. With over 300% growth in listenership and a highly engaged audience, the FYI Podcast is a tool for all those seeking to “explore the limitless world of medical informatics.”

## Supplementary Material

ooae072_Supplementary_Data

## Data Availability

The data presented in this manuscript will be shared on reasonable request to the corresponding author.
