# Supplementary material for: Empowering diversity: striving for inclusivity by leveraging the American Medical Informatics Association’s “For Your Informatics” Podcast
Source: JAMIA Open. 2024 Sep 17;7(3):ooae072. doi: 10.1093/jamiaopen/ooae072 (PMC11408726; doi:10.1093/jamiaopen/ooae072)
Supplement: ooae072_Supplementary_Data [file ooae072_supplementary_data.docx]

Supplemental Table. FYI Podcast episodes (September 2018 – June 2023) titles and length

| Episode Number | Episode Name | Length (minutes) |
| --- | --- | --- |
| 1 | Dr. Carol Friedman’s Career Journey | 28 |
| 2 | Magic in Mentoring | 31 |
| 3 | Leadership in Informatics | 24 |
| 4 | Fact and Fiction | 31 |
| 5 | Dr. Tiffani Bright’s Career path | 22 |
| 6 | Living in the Future and Out on the Edge | 31 |
| 7 | Dr. Gondy Leroy’s Career Path | 18 |
| 8 | Women in AMIA Podcast presents “XX-MEN of AMIA” Live Event | 56 |
| 9 | Dr. Peter Embi-Diversity and Inclusion in Informatics | 43 |
| 10 | Dr. Patti Brennan-Training the Next Generation of Informaticians | 45 |
| 11 | Burnout and Technology | 38 |
| 12 | Trailblazer in Dental Informatics | 46 |
| 13 | Mentoring and Networking | 38 |
| 14 | Diversifying JAMIA | 33 |
| 15 | Sponsorship & Informatics During the Pandemic | 38 |
| 16 | Engaging the Now for the Future | 24 |
| 17 | A Word from Our Sponsors: Emory School of Nursing - Project NeLL | 31 |
| 18 | Forgotten No More Series: Marty Goddard, Inventor of the Rape Kit | 30 |
| 19 | Underrepresented Leaders: Discussions with Clinical Informatics Fellowship Directors | 30 |
| 20 | AMIA as a Catalyst for Collaboration | 14 |
| 21 | History of Medical Informatics Mentorship | 30 |
| 22 | Women in Informatics Around the Globe | 23 |
| 23 | The Critical Mission to Reduce Documentation Burden | 30 |
| 24 | The Human Side of Artificial Intelligence | 65 |
| 25 | Allow Us to Reintroduce Ourselves - AMIA working groups (Part 1) | 14 |
| 26 | The Gender Pay Gap in Medicine | 36 |
| 27 | ACM-AMIA Joint Podcast Series | 37 |
| 28 | Public Health Communication + Health Informatics | 7 |
| 29 | The Informatics Industry | 42 |
| 30 | Inclusive Informatics: Reducing Bias in Our AI Systems | 63 |
| 31 | Teens, Portals, and 21^st^ Century Cures Act | 35 |
| 32 | ACM-AMIA Joint Podcast Series: Fireside Chat | 39 |
| 33 | ACM-AMIA Joint Podcast Series: Intersection of Information Systems and Medicine | 50 |
| 34 | ACM-AMIA Joint Podcast Series: Clinical AI-Challenges and Advice | 45 |
| 35 | AI in Medicine and Healthcare | 47 |
